# Combining bioinformatics techniques to explore the molecular mechanisms involved in pancreatic cancer metastasis and prognosis

**DOI:** 10.1111/jcmm.16023

**Published:** 2020-11-09

**Authors:** Jia‐Sheng Xu, Kai‐li Liao, Xinlu Wang, Jiarui He, Xiao‐Zhong Wang

**Affiliations:** ^1^ Department of vascular surgery The Second Affiliated Hospital of NanChang University NanChang China; ^2^ Department of Clinical Laboratory, Jiangxi Province Key Laboratory of Laboratory Medicine The Second Affiliated Hospital of Nanchang University Nanchang China; ^3^ Jiangxi Medical College Nanchang University Nanchang China

**Keywords:** bioinformatics analysis, differential gene, metastatic pancreatic cancer, primary pancreatic cancer, prognosis

## Abstract

This article aims to explore the underlying molecular mechanisms and prognosis‐related genes in pancreatic cancer metastasis. Pancreatic cancer metastasis‐related gene chip data were downloaded from GENE EXPRESSION OMNIBUS(GEO)database. Differentially expressed genes were screened after R‐package pre‐treatment. Functional annotations and related signalling pathways were analysed using DAVID software. GEPIA (Gene Expression Profiling Interactive Analysis) was used to perform prognostic analysis, and differential genes associated with prognosis were screened and validated using data from GEO. We screened 40 healthy patients, 40 primary pancreatic cancer and 40 metastatic pancreatic cancer patients, collected serum, designed primers and used qPCR to test the expression of prognosis‐related genes in each group. 109 differentially expressed genes related with pancreatic cancer metastasis were screened, of which 49 were up‐regulated and 60 were down‐regulated. Functional annotation and pathway analysis revealed differentially expressed genes were mainly concentrated in protein activation cascade, extracellular matrix construction, decomposition, etc In the biological process, it is mainly involved in signalling pathways such as PPAR, PI3K‐Akt and ECM receptor interaction. Prognostic analysis showed the expression levels of four genes were significantly correlated with the overall survival time of patients with pancreatic cancer, namely SCG5, CRYBA2, CPE and CHGB. qPCR experiments showed the expression of these four genes was decreased in both the primary pancreatic cancer group and the metastatic pancreatic cancer group, and the latter was more significantly reduced. Pancreatic cancer metastasis is closely related to the activation of PPAR pathway, PI3K‐Akt pathway and ECM receptor interaction. SCG5, CRYBA2, CPE and CHGB genes are associated with the prognosis of pancreatic cancer, and their low expression suggests a poor prognosis.

## 1 INTRODUCTION

Pancreatic cancer is a highly invasive malignant tumour of the digestive system with a 5‐year survival rate of <8%.^1^ Because of the earlier invasion and metastasis, the quality of life and treatment of patients with pancreatic cancer are often unsatisfactory. Therefore, in‐depth study of the specific molecular mechanism of pancreatic cancer metastasis is of vital importance for the treatment and prognosis of pancreatic cancer. Gene chip, also known as DNA chip or DNA microarray, with high flux, high integration, miniaturization, automation, etc can detect the expression levels of thousands of gene transcripts in parallel and quickly and has been widely used in mutant gene testing, differential gene screening, gene library mapping, drug targets, tumour typing, polymorphism detection, etc.^2^, ^3^ In this study, we analysed the differentially expressed genes of pancreatic cancer metastasis by analysing the data of pancreatic cancer metastasis‐related gene chip in the public database of gene chip (GEO) and analysed GO enrichment analysis, KEGG pathway analysis, protein interaction network and prognostic value analysis. And we designed experiments to verify the conclusions, providing ideas for further exploration of the molecular mechanisms of pancreatic cancer metastasis.

## 2 MATERIALS AND METHODS

### 2.1 Bioinformatics analysis of pancreatic cancer metastasis

#### 2.1.1 Materials

The three pancreatic cancer metastasis‐related gene chip data used in this study (GSE19279,^4^ GSE42952,^5^ GSE71729 ^6^) were derived from the National Bioinformatics Center (NCBI) Gene Chip Public Database (GEO).^7^ Among them, gse19279 was included in 9 cases, including 4 cases of primary pancreatic cancer and 5 cases of metastatic pancreatic cancer; gse42952 included 23 cases, including 12 cases of primary pancreatic cancer and 11 cases of metastatic pancreatic cancer; gse71729 included 206 cases, including 145 Primary pancreatic cancer and 61 metastatic pancreatic cancer.

#### 2.1.2 Method

The above data were pre‐treated and screened for differentially expressed genes by Limma method.^8^, ^9^ The screening threshold was set to *P* < 0.05, and the fold change was >2 fold. The differentially expressed genes screened by the above three data sets were taken to reduce the false positive rate; thus, the gene set obtained was used as a differentially expressed gene for pancreatic cancer metastasis for subsequent analysis. First, the DAVID online analysis software was used to perform gene function annotation and pathway analysis on differentially expressed genes of pancreatic cancer metastasis^10^, ^11^ to clarify its biological function and the involved cell signal regulation network; then use the STRING online database and Cytoscape software to perform protein interaction network analysis of pancreas Cancer metastasis differentially expressed genes,^12^, ^13^ key genes and key modules were calculated, and their possible regulation and mechanism of action were further explored. Finally, the prognostic analysis of differentially expressed genes in pancreatic cancer metastasis was performed by GEPIA online analysis tool.^14^ Prognostic analysis of the selected prognosis‐related genes was performed with data from patients with pancreatic cancer in the GEO database and KM survival curves were drawn. Further, use GEPIA online analysis tool to analyse the relationship between prognosis‐related genes and clinical stage of pancreatic cancer, so as to provide some clues for clinical prognosis.

### 2.2 Experimental verification of the selected prognosis‐related gene

The study was approved by the medical ethics committee of the First Affiliated Hospital of Nanchang University. All patients involved in the study signed the informed consent voluntarily. All methods were performed in accordance with the relevant guidelines and regulations.

#### 2.2.1 General materials

A total of 40 cases of primary pancreatic cancer and 40 cases of metastatic pancreatic cancer were selected from The First Affiliated Hospital of Nanchang University from March 2017 to May 2019. These patients were all diagnosed by CT (Computed tomography) and histopathological examination. The primary pancreatic cancer group: 28 males and 12 females, from 38 to 73 years old (mean age 51.2 ± 4.7), histological classification: 8 cases of pancreatic body and tail cancer, 32 cases of pancreatic head cancer. Metastatic pancreatic cancer group: 26 males and 14 females, from 40 to 71 years old (mean age 53.6 ± 3.9), histological classification: 7 cases of pancreatic body and tail cancer, 33 cases of pancreatic head cancer. 40 healthy people who had passed the physical examination in the Medical Examination Center, the First Affiliated Hospital of Nanchang University during the same period, were selected as the control group. Control group: 28 males and 12 females, from 35 to 76 years old (mean age 52.5 ± 3.6). There is no significance difference between the two groups of patients in gender and age (*P* > 0.05).

#### 2.2.2 Detection of expression of prognosis‐related genes in serum

Before operation, 4 mL venous blood was acquired after an overnight fast. Two hours later, the blood was centrifuged for 15 min at 1801*g*; then, serum was collected and stored in −80°C. Total DNA was extracted from 250 µL serum by using HiPure Liquid DNA Kit (purchased from Shanghai MiTuo Biotechnology Co., Ltd, shanghai, China). The expression of these four genes was detected by qPCR technique, and the primers used were shown in Table 1.

**Table 1:** PCR primers for prognosis‐related genes

Gene	Forward Primer(5′‐>3′)	Reverse Primer(5′‐>3′)
GAPDH	CTCACCGGATGCACCAATGTT	CGCGTTGCTCACAATGTTCAT
SCG5	GGGTCCTTTTGGCAACATCC	CCCCTGATCCTCACTAAAGTCC
CRYBA2	CTCTGCGCGAACCACAATG	GGGTAGTCATCAACGAGGTCAA
CPE	AGCCCTACTATCAAGGACAAACC	CCACAGCCCTTAATGGCCT
CHGB	GAGCCTCTATCCCTCCGACAG	CCCTCGCTCCCCTTTTTGA

### 2.3 Statistical analysis

SPSS 19.0 software was used for statistical analysis. All the quantitative data acquired from the experiment fitted normal distribution and could be expressed as (x ± s). The comparison between groups was conducted by single factor difference analysis and HSD‐q test. The difference is statistically significant when* P* < 0.05.

## 3 RESULTS

### 3.1 Screening of differentially expressed genes in pancreatic cancer metastasis

**Figure 1:**
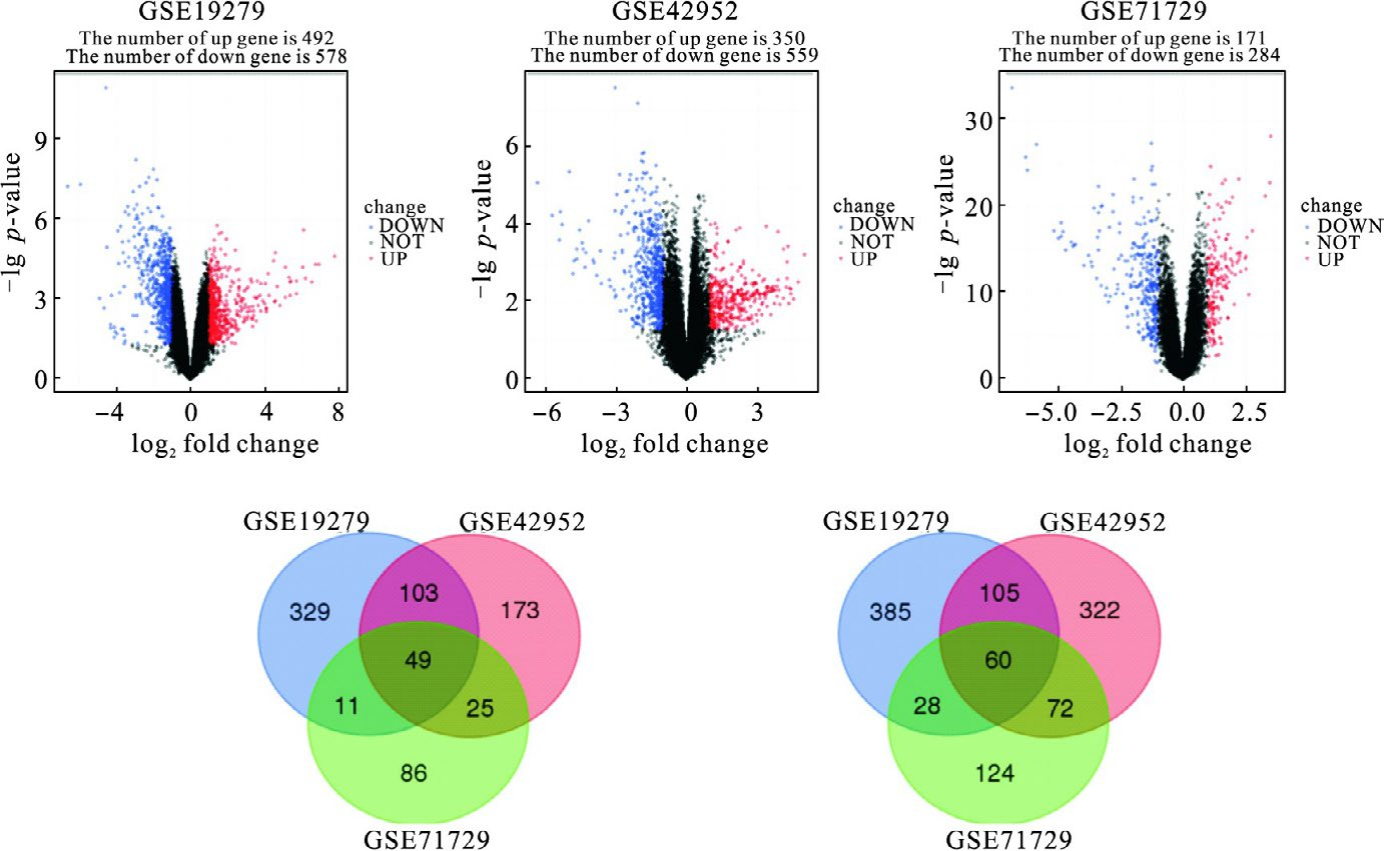
The intersection of up‐regulated and down‐regulated differentially expressed genes

Through the screening of differentially expressed genes, 1070 differentially expressed genes in the gse19279 data set were obtained, of which 492 were up‐regulated and 578 were down‐regulated. There were 909 differentially expressed genes in the gse42952 data set, of which 350 were up‐regulated and 559 were down‐regulated. There were 455 differentially expressed genes in the gse71729 data set, of which 171 were up‐regulated and 284 were down‐regulated. The three data sets were taken to obtain 109 differentially expressed genes, of which 49 were up‐regulated and 60 were down‐regulated (Figure 1).

### 3.2 Up‐regulation of differentially expressed genes by bioinformatics analysis

**Figure 2:**
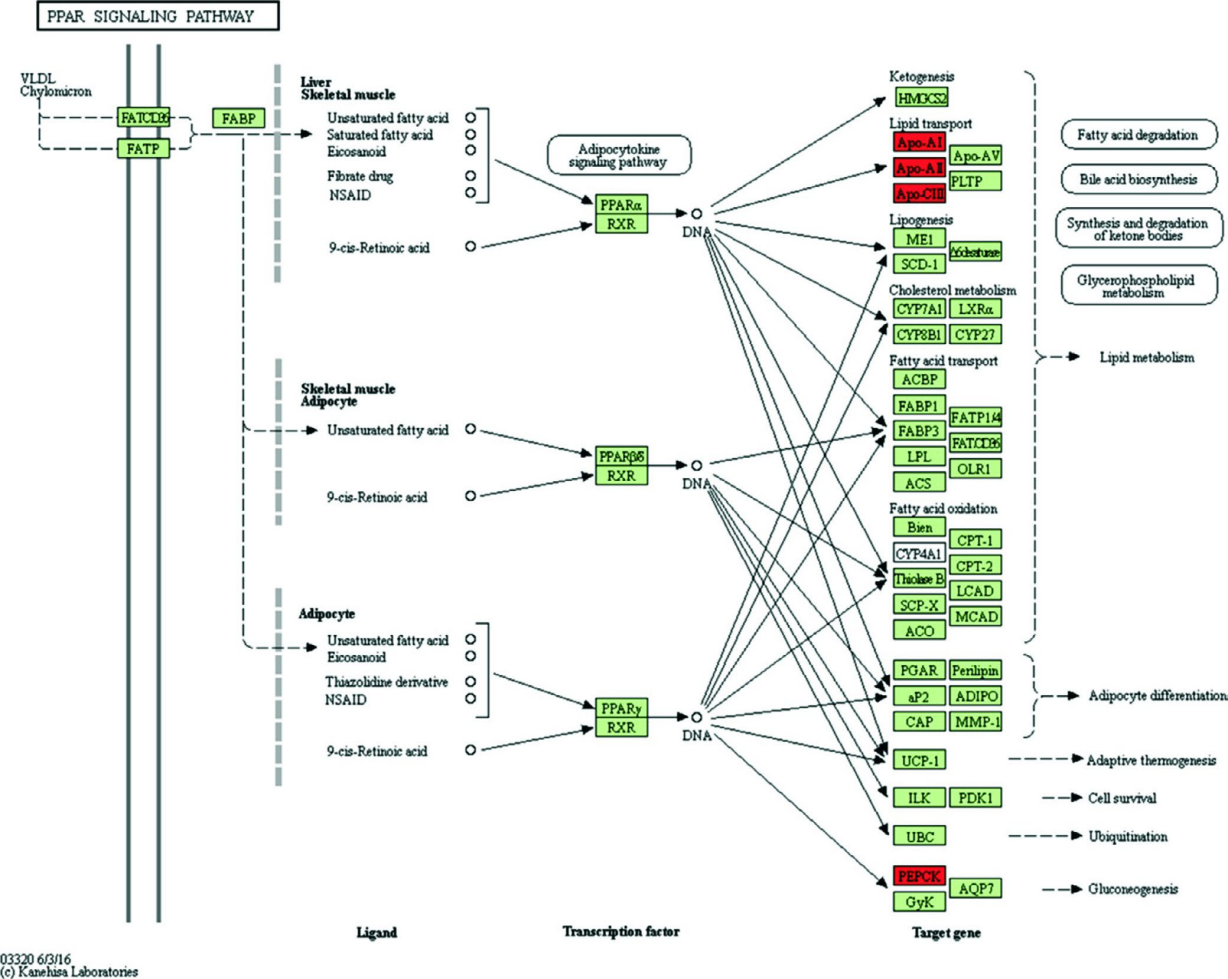
Up‐regulated differentially expressed genes participated in PPAR signalling pathway. Red: up‐regulated differentially expressed genes; green: ppar signalling pathway genes

Bioinformatics analysis of up‐regulated differentially expressed genes was performed by DAVID online analysis software. GO analysis found that these genes are mainly involved in acute inflammatory reactions, protein activation cascades, regulation of damage, negative regulation of hydrolase activity and regulation of stress and are enriched in blood particles, extracellular space, extracellular regions, extracellular exosomes and cytoplasmic membranes and bind to cellular components such as vesicle cavities, involved in enzyme inhibitor activity, endopeptidase inhibitor activity, high‐density lipoprotein particle receptor binding, lipase inhibitor activity and alcohol binding molecular function(Table 2). The KEGG pathway analysis found that it mainly focused on pathways such as complement and coagulation cascades, metabolic pathways, PPAR signalling pathways, prion diseases and amebiasis (Table 3). Up‐regulation of differentially expressed genes is involved in the PPAR signalling pathway (Figure 2).

**Table 2:** GO annotation of up‐regulated differentially expressed genes

GO ID	Term	*P*‐value	Genes
Biological process			
GO:0002526	Acute inflammatory response	1.81 × 10^−8^	AHSG, APOA2, F12, F2, HP, ITIH4, LBP, ORM1
GO:0072376	Protein activation cascade	1.81 × 10^−8^	C8A, C8G, C9, F12, F2, F9, FGB, KNG1
GO:1903034	Regulation of response to wounding	1.81 × 10^−8^	AHSG, APOA1, C8A, C8G, C9, F12, F2, FGB, HRG, KNG1, LBP, PLG, SERPINC1
GO:0051346	Negative regulation of hydrolase activity	2.36 × 10^−7^	AHSG, APOA1, APOA2, APOC1, APOC3, HRG, ITIH1, ITIH3, ITIH4, KNG1, SERPINA10, SERPINC1
GO:0006950	Response to stress	1.44 × 10^−6^	AHSG, ALB, APOA2, AQP9, ARG1, C8A, C8G, C9, CCL16, CYP2E1, F12, F2, F9, FGB, HAMP, HAO1, HP, HRG, ITIH4, KNG1, LBP, ORM1, PCK1, PLG, SERPINA10, SERPINC1
Cellular component			
GO:0072562	Blood microparticle	2.63 × 10^−26^	AHSG, ALB, APOA1, APOA2, C8A, C8G, C9, F2, FGB, HP, HPX, HRG, ITIH1, ITIH4, KNG1, ORM1, PLG, SERPINC1
GO:0005615	Extracellular space	1.33 × 10^−23^	AHSG, ALB, APOA1, APOA2, APOC1, APOC3, APOF, ARG1, C8A, C8G, C9, CCL16, F12, F2, F9, FGB, FGL1, HAMP, HP, HPX, HRG, IGFBP1, ITIH1, ITIH4, KNG1, LBP, ORM1, PLG, PON3, SERPINA10, SERPINC1
GO:0005576	Extracellular region	2.88 × 10^−16^	AHSG, ALB, APOA1, APOA2, APOC1, APOC3, APOF, ASGR1, BHMT, C8A, C8G, C9, CCL16, CFHR2, CFHR4, DPYS, F12, F2, F9, FGB, FGL1, FTCD, HAMP, HP, HPD, HPX, HRG, IGFBP1, ITIH1, ITIH3, ITIH4, KNG1, LBP, ORM1, PCK1, PLG, PON3, SERPINA10, SERPINC1
GO:0070062	Extracellular exosome	3.75 × 10^−16^	AHSG, ALB, APOA1, APOA2, APOC1, APOC3, ARG1, BHMT, C8A, C8G, C9, DPYS, F12, F2, F9, FGB, FGL1, FTCD, HP, HPD, HPX, HRG, ITIH1, ITIH3, ITIH4, KNG1, LBP, ORM1, PCK1, PLG, PON3, SERPINA10, SERPINC1
GO:0060205	Cytoplasmic membrane‐bounded	7.81 × 10^−16^	ALB, APOA1, FGB, HP, HPX, HRG, KNG1, PLG
	vesicle lumen		
Molecular function			
GO:0004857	Enzyme inhibitor activity	9.94 × 10^−7^	APOA1, APOA2, APOC1, APOC3, HRG, ITIH1, ITIH3, ITIH4, KNG1, SERPINA10, SERPINC1
GO:0004866	Endopeptidase inhibitor activity	5.04 × 10^−6^	AHSG, HRG, ITIH1, ITIH3, ITIH4, KNG1, SERPINA10, SERPINC1
GO:0070653	High‐density lipoprotein particle	1.06 × 10^−5^	APOA1, APOA2, APOC3
	receptor binding		
GO:0055102	Lipase inhibitor activity	3.04 × 10^−5^	APOA1, APOA2, APOC1, APOC3
GO:0043178	Alcohol binding	3.78 × 10^−5^	APOA1, APOA2, APOC1, APOC3, APOF, C8G

**Table 3:** KEGG pathway analysis of up‐regulated differentially expressed genes

KEGG ID	Term	*P*‐value	Genes
hsa04610	Complement and coagulation cascades	1.76 × 10^−13^	C8A, C8G, C9, F12, F2, F9, FGB, KNG1, PLG, SERPINC1
hsa01100	Metabolic pathways	.00197	ARG1, BHMT, CYP2E1, DAO, DPYS, FTCD, HAL, HAO1, HPD, MAT1A, PCK1, PON3
hsa03320	PPAR signalling pathway	.00202	APOA1, APOA2, APOC3, PCK1
hsa05020	Prion diseases	.00626	C8A, C8G, C9
hsa05146	Amoebiasis	.00643	ARG1, C8A, C8G, C9

### 3.3 Bioinformatics analysis of down‐regulated differentially expressed genes

**Figure 3:**
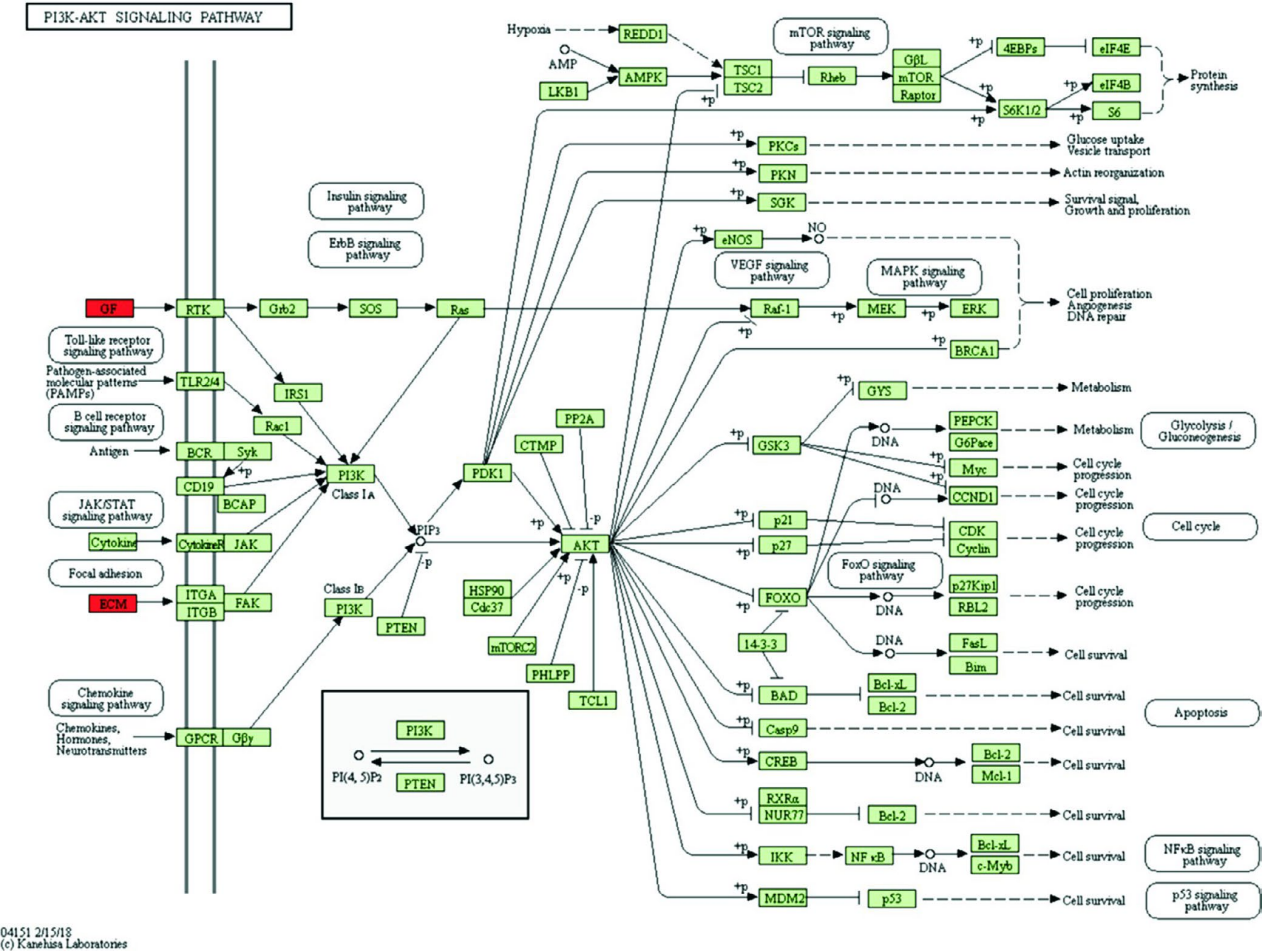
Down‐regulated differentially expressed genes participated in PI3K‐Akt signalling pathway. Red: differentially expressed genes down‐regulated; green: PI3K‐Akt signalling pathway gene

Bioinformatics analysis of down‐regulated differentially expressed genes was performed by DAVID online analysis software. GO analysis found that these genes are mainly involved in extracellular matrix construction, extracellular matrix decomposition, multicellular biocatabolism, collagen catabolism and collagen fibril construction and are enriched in the cellular components such as extracellular regions, extracellular spaces, extracellular matrix of protein cells, vesicles and endoplasmic reticulum, which are involved in molecular functions such as cell adhesion molecule binding, receptor binding, integrin binding, protein complex binding and extracellular matrix structure (Table 4).The KEGG pathway analysis found that it mainly focuses on pancreatic secretion, protein digestion and absorption, ECM receptor interaction, local adhesion and PI3K‐Akt signalling pathways (Table 5). Down‐regulation of differentially expressed genes is involved in the PI3K‐Akt signalling pathway (Figure 3).

**Table 4:** GO annotation of down‐regulated differentially expressed genes

GO ID	Term	*P*‐value	Genes
Biological process			
GO:0030198	Extracellular matrix organization	8.87 × 10^−8^	COL10A1, COL16A1, COL1A2, COL5A1, COMP, CTGF, CTRB2, CTSK, CYR61, FBN1, GREM1, LUM, SULF1
GO:0022617	Extracellular matrix disassembly	5.06 × 10^−6^	COL10A1, COL16A1, COL1A2, COL5A1, CTRB2, CTSK, FBN1, MMP11
GO:0044243	Multicellular organismal catabolic process	8.14 × 10^−6^	CEL, COL10A1, COL16A1, COL1A2, COL5A1, CTSK, MMP11
GO:0030574	Collagen catabolic process	.000116	COL10A1, COL16A1, COL1A2, COL5A1, CTSK, MMP11
GO:0 030 199	Collagen fibril organization	.000154	COL1A2, COL5A1, GREM1, LUM, MMP11
Cellular component			
GO:0005576	Extracellular region	1.16 × 10^−20^	ASPN, CDH11, CEL, CFTR, CHGB, COL10A1, COL16A1, COL1A2, COMP, CPB1, CPE, CTRB2, CTSK, CXCL14, CYR61, DKK3, DPEP1, FBN1, FCGBP, FRZB, FXYD2, GCG, GREM1, IGJ, INHBA, INS, ISLR, ITGBL1, LEFTY1, LUM, MMP11, OLFM4, PDGFD, PDGFRL, PNLIP, PPY, PRSS3, PTGDS, REG1A, REG1B, REG3A, SCG5, SCUBE2, SFRP4, SST, SULF1, THBS4, THY1
GO:0005615	Extracellular space	1.58 × 10^−15^	CEL, COL1A2, COMP, CTGF, CTRB2, CTSK, CXCL14, DKK3, DPEP1, FBN1, FRZB, GCG, GREM1, IGJ, INHBA, INS, LEFTY1, LUM, OLFM4, PPY, PRSS3, PTGDS, REG3A, SFRP4, SST, SULF1, THBS4
GO:0005578	Proteinaceous extracellular matrix	7.48 × 10^−7^	ASPN, COL10A1, COL16A1, COL1A2, COMP, CTGF, CYR61, FBN1, LUM, MMP11, THBS4
GO:0031982	Vesicle	3.05 × 10^−5^	CDH11, CEL, CFTR, CHGB, COL1A2, COL5A1, COMP, CPE, DPEP1, FBN1, FCGBP, FXYD2, GCG, IGJ, INS, ISLR, LUM, OLFM4, PDGFD, PRSS3, PTGDS, REG1A, REG1B, SCG5, STMN2, THBS4, THY1
GO:0005788	Endoplasmic reticulum lumen	6.14 × 10^−5^	COL10A1, COL16A1, COL1A2, COL5A1, GCG, INS, PDGFD
Molecular function			
GO:0050839	Cell adhesion molecule binding	4.39 × 10^−7^	COL16A1, COL5A1, CPE, CTGF, CYR61, FBN1, OLFM4, THBS4, THY1
GO:0005102	Receptor binding	7.93 × 10^−7^	CHGB, COL16A1, COL5A1, CPE, CTGF, CXCL14, CYR61, FBN1, GCG, GREM1, INHBA, INS, LEFTY1, PPY, REG1A, SST, THBS4, THY1
GO:0005178	Integrin binding	2.44 × 10^−6^	COL16A1, COL5A1, CTGF, CYR61, FBN1, THBS4, THY1
GO:0032403	Protein complex binding	2.24 × 10^−5^	COL16A1, COL5A1, COMP, CTGF, CTSK, CYR61, FBN1, IGJ, INS, LUM, THBS4, THY1
GO:0005201	Extracellular matrix structural constituent	2.40 × 10^−5^	COL1A2, COL5A1, COMP, FBN1, LUM, MFAP5

**Table 5:** KEGG pathway analysis of down‐regulated differentially expressed genes

KEGG ID	Term	*P*‐value	Genes
hsa04972	Pancreatic secretion	2.05 × 10^−6^	CEL, CFTR, CPA1, CPB1, FXYD2, PNLIP, PRSS3
hsa04974	Protein digestion and absorption	2.17 × 10^−5^	COL1A2, COL5A1, CPA1, CPB1, FXYD2, PRSS3
hsa04512	ECM‐receptor interaction	.0102	COL1A2, COL5A1, COMP, THBS4
hsa04510	Focal adhesion	.0208	COL1A2, COL5A1, COMP, PDGFD, THBS4
hsa04151	PI3K‐Akt signalling pathway	.0233	COL1A2, COL5A1, COMP, INS, PDGFD, THBS4

### 3.4 Interaction of differentially expressed genes in pancreatic cancer metastasis

**Figure 4:**
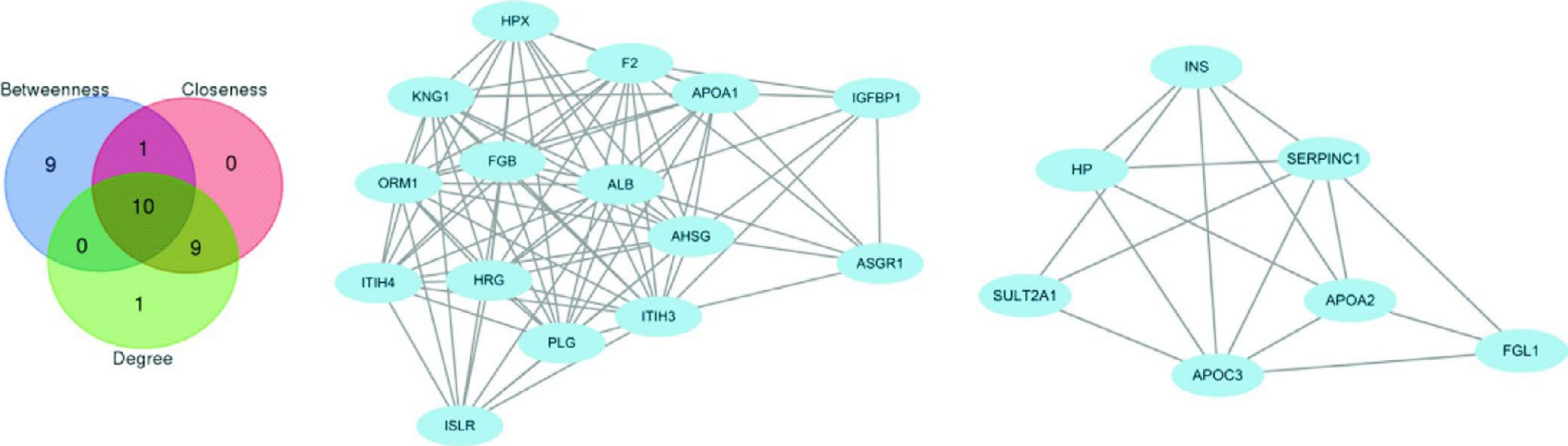
Ten key genes and two key modules

The protein interaction network of 109 pancreatic cancer metastasis differentially expressed genes was analysed by STRING online database. A total of 383 pairs of PPI relationships were obtained, which were imported into the Cytoscape computing network and the topological characteristics of each node. The results showed that the protein products encoded by the above genes were involved in the construction of protein interaction network maps, and multiple proteins were located in the centre of the network and were closely related to the surrounding proteins. Intersection was selected according to the three algorithms of Betweenness, Closeness and Degree, and the genes corresponding to 10 proteins were obtained as key genes, namely ORM1, IGFBP1, HPX, F2, APOA1, ALB, PLG, SERPINC1, KNG1 and INS, which are crucial for maintaining the network structure. Further analysis of the above PPI network resulted in two key modules with a node number greater than 5 and a threshold > 5 (Figure 4).

### 3.5 Prognosis analysis of differentially expressed genes in pancreatic cancer metastasis

**Figure 5:**
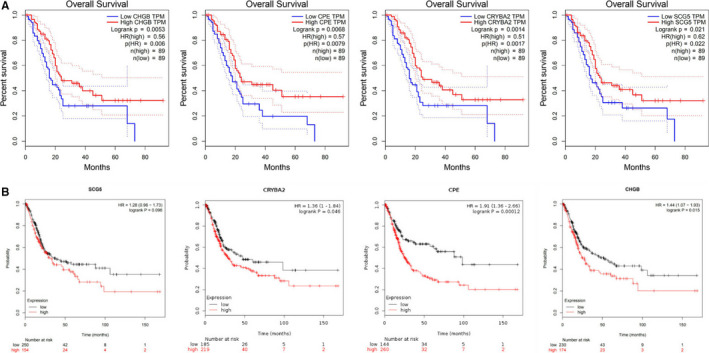
Four genes significantly associated with overall survival

**Figure 6:**
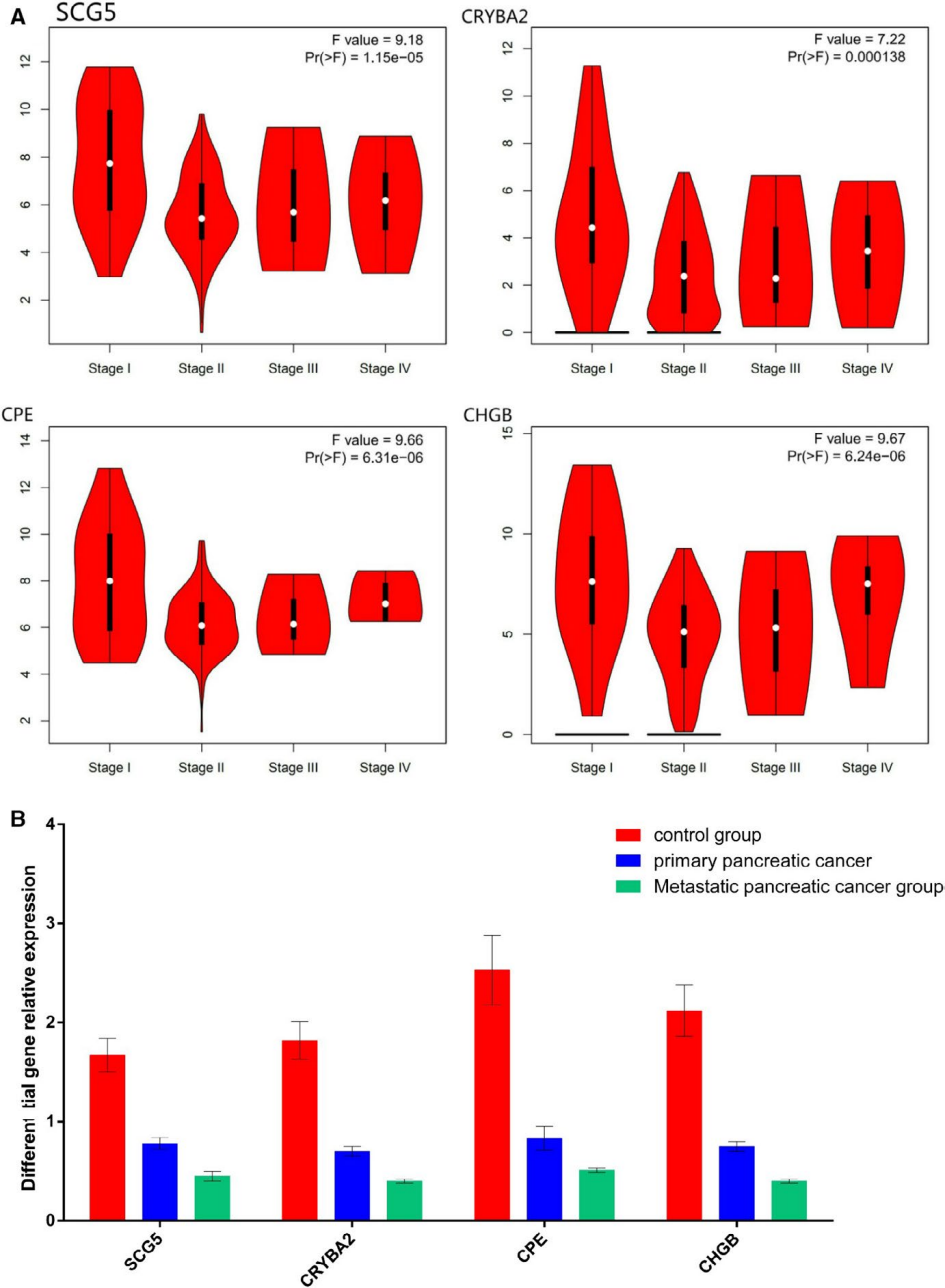
Relative expression of four prognostic‐related genes in the control group, primary pancreatic cancer group and metastatic pancreatic cancer group(Using the expression value of GAPDH as "1", calculate the relative expression levels of the four genes)

Prognostic analysis of 109 differentially expressed genes was performed by GEPIA online analysis tool. The results showed that the expression levels of four of the genes were significantly correlated with the overall survival of pancreatic cancer patients in the TCGA database, namely SCG5, CRYBA2, CPE and CHGB (Figure 5A). Using the data of pancreatic cancer patients in the GEO database to verify the relationship between the expression of four differential genes and prognosis, the KM survival curve was drawn (Figure 5B), and the results were consistent with the TCGA data analysis. The relationship between the expression of four prognosis‐related genes and the clinical stage of pancreatic cancer is shown in Figure 6A.

### 3.6 Experimental verification of differential genes

Compared with the control group, the expression levels of the four genes in the primary pancreatic cancer group and the metastatic pancreatic cancer group were significantly decreased. The relative expression levels of the four genes in metastatic pancreatic cancer were lower than those in primary pancreatic cancer, and the difference was significant (*P* < 0.05) (Figure 6B).

## 4 DISCUSSION

Metastasis is an important risk factor for poor prognosis in patients with pancreatic cancer. Because patients with metastases often lose their chance of surgery, they can only rely on palliative care.^15^ At present, the specific molecular mechanism of pancreatic cancer metastasis has not been fully elucidated, which involves the abnormal expression of many genes and the imbalance of related signalling pathways. Therefore, studying pancreatic cancer metastasis‐related genes, and then revealing the nature of pancreatic cancer metastasis at the molecular level, has become one of the hotspots of current pancreatic cancer research. Gene chip technology is a high‐throughput, fast and accurate method for analysing gene expression. It can detect gene chips under different conditions, different cell types, different intervention methods, different cell growth stages and states and produce massive amounts of data. The data enable researchers to use bioinformatics methods to integrate analysis and explore potential biological information, thus providing useful clues for exploring the molecular mechanisms of pancreatic cancer metastasis.

In this study, 109 genes of differentially expressed genes were found by analysing the gene chip data (GSE19279, GSE42952, GSE71729) related to pancreatic cancer metastasis in the GEO database, of which 49 were up‐regulated and 60 were down‐regulated. In order to further clarify the biological functions of these differentially expressed genes, GO enrichment analysis and KEGG pathway analysis were performed with DAVID online software. GO analysis showed that up‐regulated differentially expressed genes are mainly involved in acute inflammatory reactions, protein activation cascades, regulation of damage, negative regulation of hydrolase activity, and regulation of stress, and other biological processes and are enriched in blood particles, extracellular space, extracellular regions, extracellular exosomes, and cytoplasmic membrane‐bound vesicles and other cellular components, involved in molecular functions such as enzyme inhibitor activity, endopeptidase inhibitor activity, high‐density lipoprotein particle receptor binding, lipase inhibitor activity and alcohol binding; down‐regulated differentially expressed genes are mainly involved in extracellular matrix construction, extracellular matrix decomposition, multicellular biocatabolism, collagen catabolism and collagen fibril construction and are enriched in the cellular components such as extracellular regions, extracellular spaces, extracellular matrix of protein cells, vesicles and endoplasmic reticulum, which are involved in molecular functions such as cell adhesion molecule binding, receptor binding, integrin binding, protein complex binding and extracellular matrix structure. The results of this analysis suggest that the above biological processes may play an important role in the metastasis of pancreatic cancer. The results of KEGG analysis show that up‐regulated differentially expressed genes are mainly concentrated in the complement and coagulation cascades, metabolic pathways, PPAR signalling pathways, prion diseases and amebiasis. Down‐regulated differentially expressed genes are mainly concentrated in pancreatic secretion, protein digestion and absorption, ECM receptor interaction, local adhesion and PI3K‐Akt signalling pathway. The role of PPAR signalling pathway in pancreatic cancer metastasis has been reported in the literature. Li et al^16^ have reported that activation of PPARγ can up‐regulate the tumour suppressor gene PTEN, thereby inhibiting the expression of MMP‐2, thus impairing the migration and invasion of pancreatic cancer cell lines. As the PI3K‐Akt signalling pathway is one of the star signalling pathways for extensive researches, its abnormal activation for promoting invasion and metastasis of pancreatic cancer has been confirmed by many studies.^17^, ^18^ Inhibition of abnormal activation of PI3K‐Akt signalling pathway has become one of the strategies for inhibiting the metastasis of pancreatic cancer.^19^

Based on the STRING online database, we performed a protein interaction network analysis on differentially expressed genes in pancreatic cancer metastasis. It found that the protein products encoded by the above genes are involved in the construction of protein interaction network maps, which are connected by wires and some genes are used as centres and are closely related to the surrounding gene products. In this study, 10 key genes in the network were screened by Betweenness, Closeness and Degree. They were ORM1, IGFBP1, HPX, F2, APOA1, ALB, PLG, SERPINC1, KNG1 and INS. Among them, IGFBP1 is a member of the insulin‐like growth factor binding protein gene family. Thakur et al^20^ have reported that liver metastases of pancreatic cancer show high expression of IGFBP1 compared with the primary tumour. In addition to pancreatic cancer, IGFBP1 has been reported to be closely associated with metastasis in several other common digestive system tumours. Gong et al^21^ have found that inhibition of FASN in hepatocellular carcinoma cell lines reduces the migration and invasion by reducing the HIF‐1α/IGFBP1 pathway. Kim et al^22^ also confirmed that overexpression of IGFBP1 promotes liver metastasis in nude mice experiments with colorectal cancer cell lines. The above studies demonstrate that IGFBP1 plays an important role in tumour metastasis, which is consistent with the results obtained in this study. However, several other key genes are currently lacking sufficient literature support, and more relevant research will be needed in the future.

Prognostic analysis of 109 pancreatic cancer metastasis differentially expressed genes was performed by GEPIA online analysis tool. The results show that the expression levels of four genes were positively correlated with the overall survival time of pancreatic cancer patients in the TCGA database, namely SCG5, CRYBA2, CPE and CHGB, which are protective factors for the prognosis of pancreatic cancer. These results suggest that the abnormal expression of the above genes is closely related to the metastasis and prognosis of pancreatic cancer and thus has certain clinical significance for the judgement of the progression and prognosis of pancreatic cancer.

## 5 CONCLUSION

In summary, this study used an integrated bioinformatics method to comprehensively analyse the data of pancreatic cancer metastasis‐related gene chips in the GEO database and successfully screened 109 differentially expressed genes, which were analysed by GO enrichment analysis and KEGG pathway analysis and obtained a series of related biological processes and signalling pathways, providing a theoretical basis for laboratory research on pancreatic cancer metastasis. In addition, by constructing a protein interaction network map, two key modules and 10 key genes were obtained, and the study of IGFBP1 suggested its important position in tumour metastasis. In addition, by prognostic analysis of the above differentially expressed genes, four genes with significant prognostic significance were obtained, which is important for evaluating and predicting the prognosis of patients with pancreatic cancer.

## CONFLICT OF INTEREST

The authors confirm that there are no conflicts of interest.

## AUTHOR CONTRIBUTION

**Jiasheng Xu:** Conceptualization (lead); Writing‐original draft (lead). **Kaili Liao:** Resources (lead); Writing‐review & editing (supporting). **Xiaozhong Wang:** Funding acquisition (lead); Writing‐review & editing (supporting). Xinlu Wang:revised the manuscript (supporting). Jiarui He: revised the manuscript (supporting).

## 6 ETHICAL APPROVAL AND CONSENT TO PARTICIPATE

The study was approved by the medical ethics committee of the First Affiliated Hospital of Nanchang University. All patients involved in the study signed the informed consent voluntarily. All methods were performed in accordance with the relevant guidelines and regulations.

## Data Availability

Data sharing not applicable to this article as no data sets were generated or analysed during the current study.
